# Opportunities for Improved Chagas Disease Vector Control Based on Knowledge, Attitudes and Practices of Communities in the Yucatan Peninsula, Mexico

**DOI:** 10.1371/journal.pntd.0002763

**Published:** 2014-03-27

**Authors:** Kathryn Rosecrans, Gabriela Cruz-Martin, Ashley King, Eric Dumonteil

**Affiliations:** 1 Laboratorio de Parasitología, Centro de Investigaciones Regionales “Dr. Hideyo Noguchi”, Universidad Autónoma de Yucatán, Mérida, Yucatán, Mexico; 2 Department of International Health, School of Public Health and Tropical Medicine, Tulane University, New Orleans, Louisiana, United States of America; 3 Department of Tropical Medicine, School of Public Health and Tropical Medicine, Tulane University, New Orleans, Louisiana, United States of America; Universidad de Buenos Aires, Argentina

## Abstract

**Background:**

Chagas disease is a vector-borne parasitic disease of major public health importance. Current prevention efforts are based on triatomine vector control to reduce transmission to humans. Success of vector control interventions depends on their acceptability and value to affected communities. We aimed to identify opportunities for and barriers to improved vector control strategies in the Yucatan peninsula, Mexico.

**Methodology/principal findings:**

We employed a sequence of qualitative and quantitative research methods to investigate knowledge, attitudes and practices surrounding Chagas disease, triatomines and vector control in three rural communities. Our combined data show that community members are well aware of triatomines and are knowledgeable about their habits. However, most have a limited understanding of the transmission dynamics and clinical manifestations of Chagas disease. While triatomine control is not a priority for community members, they frequently use domestic insecticide products including insecticide spray, mosquito coils and plug-in repellents. Families spend about $32 US per year on these products. Alternative methods such as yard cleaning and window screens are perceived as desirable and potentially more effective. Screens are nonetheless described as unaffordable, in spite of a cost comparable to the average annual spending on insecticide products.

**Conclusion/Significance:**

Further education campaigns and possibly financing schemes may lead families to redirect their current vector control spending from insecticide products to window screens. Also, synergism with mosquito control efforts should be further explored to motivate community involvement and ensure sustainability of Chagas disease vector control.

## Author Summary

Chagas disease is an important parasitic disease transmitted by triatomine bugs. Current prevention efforts are based on eliminating triatomines from homes to reduce disease transmission to humans. However, the success of these control interventions depends on their acceptability and value to affected communities. We aimed to identify opportunities for and barriers to triatomine control strategies in the Yucatan peninsula, Mexico. We used a sequence of group discussion, interviews, and a survey to investigate the perception and knowledge of communities on Chagas disease and triatomines in three villages from the Yucatan peninsula, Mexico. Inhabitants are rather familiar with triatomine bugs, but do not associate well these bugs with Chagas disease and its clinical manifestations. Mosquito rather than triatomine control is a common preoccupation, and households frequently use insecticide spray, mosquito coils and plug-in repellents, spending about $32 US per year on these products. Alternative methods such as yard cleaning and window screens are perceived as desirable and potentially more effective. Screens are nonetheless described as unaffordable. The promotion of education campaigns and possibly financing schemes could help families to redirect their current spending from insecticide products to window screens. Also, synergism with mosquito control efforts should be further explored to motivate community involvement and ensure sustainability of Chagas disease vector control.

## Introduction

Chagas disease is a vector-borne parasitic disease endemic in the Americas, where it is of major public health importance, affecting up to 8–9 million people [1]. Recent estimates also illustrate the very high disease burden it causes [2]. Chagas disease is present in Mexico, but its importance is poorly documented and controversial. While the Ministry of Health officially reports a few hundred cases [3], one study estimated the number of people infected to be nearly 6 million [4]. The only nationwide studies are from the late 1980s and indicate a national seroprevalence of 1–2%, with an important heterogeneity among regions and states [5]–[7]. In Yucatan, seroprevalence ranges from 1% in urban areas up to 5% in rural villages [8], [9]. Despite these data, there is no national vector control program to reduce transmission, nor are there education campaigns to raise awareness of the disease in Mexico.

The Mexican health care system is divided into the *Instituto Mexicano de Seguro Social* (IMSS), which covers workers in the private sector, and the *Instituto de Seguridad y Servicios Sociales de los Trabajadores* (ISSSTE), which covers workers in the public sector. The *Seguro Popular de Salud*, launched in 2004, aims at covering the 48 million the lowest income quintile left uninsured by IMSS and ISSSTE programs [10]. These programs provide basic health care and medicine to patients, and some health education. Vector control activities, mostly focused on the prevention of dengue, and to a lesser extent on malaria, are conducted directly by the *Secretaria de Salud*. The current national guidelines [11] recommend the cleaning of the house in case of triatomine infestation, followed by insecticide spraying in case of recurrent infestation or if a human case is detected, and are applied on a case by case basis.

Most vectorial transmission to humans is associated with domiciliated triatomines species well adapted to human housing. However, several autochtonous triatomine species can transiently invade houses and present different degrees of adaptation to human housing. In these conditions, conventional indoor insecticide spraying is of limited efficacy for vector control, and it is thus necessary to design novel vector control interventions that are tailored to vector behavior and ecology for better efficacy [12]–[15].

Communities' knowledge, attitudes and practices related to vector-borne diseases vary depending on the vector and may moderate acceptance of and participation in vector control activities [16]–[19]. For example, while conventional insecticide spraying of domiciles for triatomine control was well-accepted by communities in Honduras, an alternative based on insecticidal paint had a low level of acceptance from both the communities and vector control personnel because it was perceived as less effective and had a strong odor [20]. As success of vector control interventions depends on their acceptability and value to affected communities, an understanding of sociocultural factors is needed to inform development of control strategies [21].

Communities affected by Chagas disease often have biological and ecological knowledge of triatomine vectors, while understanding of parasite transmission and the disease itself is more limited [22]–[27]. Community members' knowledge and perceptions can however be quite variable, depending on the epidemiological importance of Chagas disease in the area, and the intensity of previous vector control and education activities [23], [24], [26], [28], [29]. Common community practices and public health interventions to control triatomine house infestation also vary, from a heavy reliance on insecticide spraying in Honduras [23], to a focus on improved hygiene and housing improvement in Minais Gerais, Brazil [24] and Bolivia [25].

In the Yucatan peninsula, Mexico, *Triatoma dimidiata* is the main vector and infests houses on a seasonal basis [30]–[38]. Some risk factors for house infestation have been identified [34], [39], [40] and may be targeted for improved control. Integrated vector control interventions have been evaluated [14], [15], [41], however, no information is available on how communities perceive the disease and *T. dimidiata* vectors. In the present study, we investigated community knowledge, attitudes and practices surrounding Chagas disease, triatomines and vector control to identify opportunities for and barriers to improved vector control strategies, as well as needs for tailored Chagas disease education.

## Materials and Methods

### Study sites

**Figure 1:**
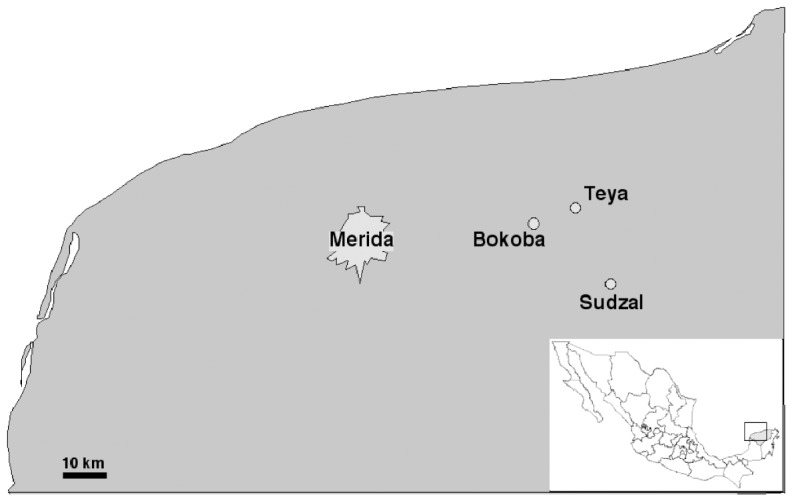
Map of the study area.

The study was carried out in the villages of Bokoba (21.01°N, 89.07°W), Teya (21.05°N, 89.07°W) and Sudzal (20.87°N, 88.98°W), located about 15–20 km apart in the central part of Yucatan, Mexico (Figure 1). There are a total of 570, 702 and 416 houses in Bokoba, Teya and Sudzal, respectively, all of which have been georeferenced [34]. The respective populations are of about 2,000 inhabitants in both Bokoba and Teya and 1,600 in Sudzal, with about 40% of the population below 14 years of age [39]. Most of the population (over 90%) are native Mayan speakers (most are also native Spanish speakers). Most heads of households are subsistence farmers (38%), and a few work in construction or manufacture (14%); a minority have a regular work contract (22%) [39]. The majority (63%) have limited income and receive social welfare benefits (“*Oportunidades*” program) [39]. The average educational level is completion of primary school [39]. Triatomine surveys and Chagas disease-related research activities have been carried out in these communities since 2006, including a pilot vector control intervention [14] and a serological survey, as well as basic Chagas disease education and awareness.

### Study design

A sequence of qualitative and quantitative research methods was employed to investigate community members' knowledge, attitudes and practices surrounding Chagas disease, triatomines and vector control in each of the study communities. Freelisting, the first method, provided us with appropriate terminology and an entry point to engaging in further research in the communities [42]. It also allowed us to evaluate triatomines and Chagas disease in reference to other biting insects and vector-borne diseases to gain greater perspective on participant priorities and evaluate the potential for synergistic, multi-disease vector control interventions. Results from freelisting were used to develop the content of ranking exercises, which in turn informed development of the focus group discussion guide. We explored threat perception, as defined in the Health Belief Model as perceived severity and susceptibility, by asking about dangerousness of biting insects and biting frequency [43]. Results from all previous methods were used to design a survey to quantify and further explore previous results. All fieldwork was performed by experienced social scientists. This mixed methods approach allowed us to leverage the comparative strengths of qualitative and quantitative approaches and to use complementary data to triangulate findings and corroborate conclusions [44].

#### Freelisting and ranking exercises

Freelisting was conducted as described by Weller and Romney [45] and Bernard [46]. A convenience sample of community members from each village was asked to list either biting insects or insect prevention methods. Prompts were used until participants could name no additional insects or prevention methods. We calculated the frequency with which each insect prevention method was mentioned as well as each item's salience score, a measure that takes into account both the frequency with and the order in which items are mentioned [45].

Using the freelisting results, ranking cards were created with the names and pictures of the 12 most commonly mentioned biting insects from the freelisting exercise [46]. Participants were asked to order the cards from most to least dangerous. Different participants were asked to rank the same insects in order from those that bite most frequently to those that bite least frequently. Participants were also asked to rank ten cards depicting methods of insect prevention identified during the freelisting exercise from most to least effective. For ranking exercises, we calculated both the average rank and a weighted rank using the cultural consensus model [47], [48]. To determine the average rank, we summed the rank order for each item and divided by the number of respondents. We then ordered them, with the item with the lowest average having the highest rank. To calculate the weighted rank, we calculated a cultural competency score for each respondent by comparing rank agreement between that respondent and each other respondent for each item [47], [48]. Respondents that had higher agreement were considered to be more competent in the ranking domain. Responses were then weighted with each respondent's cultural competency score and the average rank was calculated in the same manner as above. This method takes into account variable domain knowledge among respondents.

#### Focus groups

Research team members moderated six sex-segregated focus groups (three each of men and women) of three to ten people in each study village. A local facilitator from each village invited community members to participate, organized meetings, and took notes during the discussions, which were also digitally recorded. Moderators used a discussion guide to explore themes including biting insects and their habits, diseases associated with insect bites, and methods and strategies to prevent insect infestation and bites. Participants were not asked directly about triatomines or Chagas disease, but asked about biting insects and then questioned about associated diseases. Attitudes toward potential Chagas disease vector control interventions, including window insect screens and insecticide-impregnated curtains, were also investigated.

Focus group recordings were transcribed and then coded by a member of the research team, using thematic analysis [49]. Briefly, transcripts were used to generate a codebook, which was in turn used to identify themes based on both semantic content and interpretations [49]. Themes were then refined to produce a thematic map. Finally, we selected representative participant quotations to exemplify relevant themes.

#### Survey

Freelisting, ranking, and focus groups findings were used to develop a two-part survey, which was applied between October 2010 and November 2011 by teams of two field workers trained in administering the survey [39]. Where possible, wording of standard questions was taken from national surveys. Questions pertaining to triatomines, Chagas disease, and insect prevention were drafted with language used by participants in the focus group discussions. Questions either stated the possible responses or were open-ended but categorized by data collectors at the time of response. The survey was pilot-tested in a nearby community that was not involved in the study, and questions were revised for clarity.

We first randomly selected 345 households using Excel's random number generator function (155, 111, and 79 from Teya, Bokoba and Sudzal, respectively) and invited them to participate in the survey. Data were collected from 308; the remaining 37 households were abandoned houses, declined to participate, or those in which inhabitants were unavailable after three visits. Results of the household portion of the survey are reported elsewhere [39]. Using pre-printed Kish tables, one adult was selected from each household and invited to complete an additional individual-level survey on knowledge of triatomines, Chagas disease, and attitudes towards insect control methods. Data from three hundred and eight households were included in the analysis and two hundred thirty nine participants completed the individual portion of the survey. Some participants did not answer every questions, but no data were excluded from the analysis. The remaining individuals either declined to participate or were unavailable after three visits.

For quantitative analysis of survey data, frequencies were calculated and compared among different groups of respondents using χ^2^ tests. Continuous variables are presented as mean and SD or as box plots showing the median, 25^th^ and 75^th^ percentiles and minimum and maximum values. For estimates of household spending on different products for vector control, we used recalled spending from the previous week (mosquito coils and plug-in repellent), the previous month (insecticide spray and repellent skin cream) or the previous year (professional insecticide spraying and herbicide). As survey data were collected over an entire year, we assumed that reported spending was not representative of a specific season, but rather provided an idea of average spending throughout the year. We calculated the cumulative annual spending per household based the recalled spending for each product.

### Ethics statement

The protocol and all activities were reviewed and approved by both the World Health Organization and the Autonomous University of Yucatan institutional bioethics committees. All participants provided written informed consent before engaging in any research activities.

## Results

### Knowledge and threat perception of biting insects

#### Freelisting and ranking exercises

When asked to list insects that bite people in their villages, 76% of participants cited triatomines, referred to locally as the “*pic*” in Mayan; only mosquitoes were mentioned more frequently (87%). This remained true when the order in which insects were listed was taken into account (with those mentioned first weighted more) using the salience score [45] (Supplementary table S1). Participants also mentioned the “chinche,” which through later focus group discussions was found to be variously understood as a large group of bugs that includes the *pic*, another word for *pic*, or a bug similar to, but different from, the *pic*.

Table 1 shows the average rank of dangerousness and biting frequency of insects, as well as the cultural consensus weighted rank. *Triatomines* ranked near the top, suggesting a high threat perception. The cultural consensus model showed high agreement among participants regarding the dangerousness of insects, and marginal agreement regarding biting frequency.

**Table 1:** Relative dangerousness and biting frequency of common insects.

	Dangerousness (n = 45)		Biting frequency (n = 44)	
	Average rank	Cultural consensus rank	Average rank	Cultural consensus rank
1	Snake	Snake	Mosquito	Mosquito
2	Tarantula	Tarantula	Scorpion	“Pic”
3	“Pic”	“Pic”	“Pic”	Scorpion
4	Scorpion	Scorpion	Bee	Bee
5	Mosquito	Mosquito	Ant	Horsefly
6	Bee	Bee	Horsefly	Ant
7	Cockroach	Cockroach	Cockroach	Wasp
8	Wasp	Wasp	Wasp	Cockroach
9	Spider	Spider	Tick	Tick
10	Tick	Tick	Tarantula	Snake
11	Horsefly	Horsefly	Snake	Tarantula
12	Ant	Ant	Spider	Spider

#### Focus groups

Both male and female focus groups spoke about mosquitoes when asked about biting insects. They are the most frequent biters and, additionally, are the most annoying. Participants complained that the mosquitoes kept them up at night, both because of their evening biting habits and the noise they make flying around.

Every focus group mentioned triatomines, if not spontaneously, then when prompted to name more biting insects. Very few individuals said they were unfamiliar with the bug. Many recounted stories of finding a triatomine behind furniture or under things. Both men and women said that when they found a triatomine in the house, they killed it, usually with a flip-flop. When asked, the majority said that the triatomines are active at night and several described them entering the house through a window rather than living in the house. Indeed, triatomines were mostly associated with peridomestic or sylvatic habitats, hiding under rocks or in dirty peridomiciles.

M1: “Under rocks in the bushes. Also in houses where there is lots of trash… under rocks. Yes, where there is lots of trash, cardboard.” (See Supplementary text S1 for original quotes in Spanish).

F1: “Yes, for example if we collect rocks and make a pile and sometimes they make their little nest there and live there.”

F2: “The *pic* is during the night, because during the night is when they come inside to bite.”

The threat posed by triatomines and their relationship with Chagas disease was much less straightforward for community members. Both men and women said that a triatomine bite causes swelling and sometimes itches. They mentioned that it could also cause bruising or produce pus. More women than men volunteered information on treating triatomine bites. Women said that one should put salt and/or lemon on the wound to help it heal. Infection was mentioned as a possible complication of the bite. A few suggested that one should go to the health center if bitten by a triatomine. More women than men mentioned having learned about triatomines at a meeting at a school, the town hall, or the health center. Some recalled that the insect could carry a disease, but few remembered the name of the disease. Various descriptions of disease were given, from previously mentioned swelling, to skin infection, cancer, and heart problems. Some said that the disease took a while to develop. Importantly, all descriptions of disease and risk associated with triatomines were not expressed as truly owned, but rather as something that they had been told or had heard, without really being sure if they should believe it nor not.

M2. “It is real bad, the *pic* is real bad, it transmits, I don't know what, a disease…”

M3. “When it bites you it get swollen and it stays like this… With time it is the reaction, right away you cannot see, you cannot see if it can give you some disease, but with time yes, you can detect the disease, they say that it is of the heart.”

F3. “It produces cardiac arrest, this is what they say, this is what I heard in the talk.”

F4. “Because once it bites you, it will always give you the Chagas, because the Chagas they say it is a microbe that you cannot see.”

F5. “They say that it damages the heart and all your organs, it makes them age, that's how it kills you, it absorbs the blood from your heart, and they say that it's called Chagas this disease.”

Dengue fever came up much more frequently as a disease transmitted by insects and particularly mosquitoes, and prompted discussion of mosquito control measures such as emptying water receptacles.

#### Survey

Knowledge of triatomines was generally good (Table 2). When showed photos of several insects, 93% (195/210) correctly identified the picture of an adult triatomine. Fewer (36%, 76/210), however, correctly identified the photo of a triatomine nymph. When asked what triatomines eat, 73% (160/219) said blood. Half of participants said that triatomines were more active at night.

**Table 2:** Quantitative survey of knowledge, attitudes and practices surrounding Chagas disease and triatomines.

	%	N
**Triatomine knowledge**		
Know insect called “pic”	94	226
Identified photo of adult triatomine	93	210
Identified photo of triatomine nymph	36	210
Triatomines active at night	50	221
Triatomines feed on blood	73	219
**Chagas disease knowledge**		
Triatomine bite leads to Chagas disease	8	137
Triatomine bite leads to a disease/illness	52	183
Triatomine bite swells and/or forms pus	61	195
Triatomine bite leads to heart attack	15	142
Disease transmitted by triatomine affects heart	38	147
Disease transmitted by triatomine takes years to develop	35	162
**Threat perception**		
Triatomine is more dangerous than mosquito and cockroach	75	221
Disease transmitted by triatomine is more dangerous than dengue fever and influenza	20	164
Very probable that someone in the household could be bitten by triatomine	23	218
Very probable that someone in the household could get sick from triatomine bite	30	170
**Experience with triatomines**		
Have seen triatomine in house	71	220
Have been bitten by triatomine	38	213
Have brought triatomine to health center	37	222
Learned about triatomines at health center	58	153

Comparatively, and in agreement with qualitative data, knowledge about Chagas disease was rather low. About half (52%, 95/183) of participants said that triatomines can cause disease, but only 8% (11/137) specifically named Chagas disease. It should be noted that, as used during focus groups, the word “disease” may sometimes refer to the bite wound itself. Only 15% (21/142) of participants spontaneously said that the disease caused by triatomines can lead to a heart attack, but when asked what part of the body can be affected by the disease, 38% (56/147) identified the heart.

The triatomine itself is considered dangerous, but this may be due to the severity of the acute bite rather than the possibility of chronic disease. The triatomine is seen as more dangerous than a mosquito or cockroach (75%, 166/221), but the disease caused by triatomines is not seen as more dangerous than dengue fever or influenza (20%, 33/164). Less than a third of participants considered it very likely that someone in their household might be bitten by a triatomine (23%, 50/218) or contract a disease from a triatomine (30%, 51/170). Again, this is consistent with the results of the focus groups, indicating an awareness that triatomines can bite, but a less straightforward understanding of the underlying pathology of Chagas disease or its potential severity.

Most (71%, 156/220) have seen triatomines in their homes, and 38% (81/213) said they had been bitten by a triatomine before. Thirty-seven percent (82/222) said that someone in their household had brought a triatomine to the health center as part of the entomological surveillance program implemented in the communities. More than half of those who knew about triatomines and Chagas disease (58%, 89/153) had learned about triatomines at the health center.

### Prevention and vector control methods used

#### Freelisting and ranking exercises

Aerosol (spray can) insecticide was mentioned most frequently as a method to prevent insects in the house, followed by two methods specifically targeting mosquitoes, mosquito coils and plug-in mosquito repellent (Supplementary Table S2). Patio cleaning and window or door screens, both methods that have been shown to have a protective effect against triatomine infestation in Yucatan, were also mentioned. Participants listed fewer insect prevention methods than bugs, and only aerosol insecticide was named by more than half of the participants. In contrast, the five most frequently cited bugs were mentioned by more than half of participants.

Participants ranked cards depicting methods of insect prevention from most to least effective (Table 3). However, cultural consensus analysis yielded insufficient agreement, indicating that there is no consensus on which methods of insect prevention are most effective.

**Table 3:** Relative effectiveness of insect prevention methods.

Average rank (most to least effective)	Prevention method
1	Aerosol insecticide
2	Patio cleaning
3	House cleaning
4	Window/door screens
5	Mosquito coil
6	Plug-in mosquito repellent
7	Bed/hammock net
8	Brush/plant burning
9	Herbicide
10	Insect repellent skin cream

Based on N = 44 individuals in ranking exercise.

#### Focus groups

Both men and women said that women were primarily responsible for insect control measures in their household, and in all cases, the unanimous motivation for actions (which were focused on mosquitoes) was to protect their children. Aerosol insecticide is often sprayed into the air. Some said that they spray the insecticide, close the doors and leave the house for a period of time. Many described measures they (or their wives or mothers) take to prevent the mosquitoes from coming in the house, in some cases daily, including: shutting all windows and doors early in the evening, lighting mosquito coils, and using plug-in mosquito repellents. Some participants said they could not use the mosquito coils because they caused coughing or difficulty breathing for themselves or another family member. Usually, someone in the group made a comment about the impossibility of getting rid of mosquitos. Preventative methods are either not effective or work only temporarily; some mosquitoes always sneak by.

F6. “I spray insecticide, most of the time I spray because they [mosquitoes] all fall dead and don't stay around.”

In some groups yard cleaning was mentioned spontaneously as an insect control measure. In other groups, it was only brought up after prompting. Yard cleaning consists of collecting trash and cutting down plants and grass. It sometimes also includes burning trash, which is said to drive insects away. Many men said that they were the ones that cleaned the yards at their houses, but when female focus groups were asked they often laughed and said that women are the ones that do it. They said that often the men do not have time because they are away working. Both male and female groups said that some people do not keep their yards clean either out of laziness or because of lack of time. Some found it pointless to clean their own yard if their neighbor did not maintain his yard clean.

M4. “Clean the vegetation, like for example the weeds, the trash, the rocks, collect them together so that bugs don't have a place to hide.”

M5. “Like most bugs, because when they see it's clean they don't like it, they like it where there is trash.”

Some participants had screens in some or all of their windows, but they described screens of different quality. Plastic screens were said to be cheaper, but metal screens last longer. Some had installed the screens themselves, usually nailing or otherwise attaching them to the outside of the window. Others had had someone professionally install them with frames. Distinction was made between wooden frames and metal ones sealed with rubber, the latter said to be more effective. Women usually gave more details about the screens. Both men and women thought that window screens were desirable. The only but significant barrier was cost. When asked if they would install screens in their windows, most said it depended on the cost. Women said that if the cost were significant, they would have to consult with their husbands before proceeding.

M6. “Mosquito screens. Yes they are very good because if it is hot you can open your door and mosquitos don't come in.”

F7. “For me the best is, having mosquito screens, because these really prevent them from entering.”

F8. “Either we put mosquito screens or we eat.”

F9. “But sometimes the economy is not here to put mosquito screens like (F3) said, sometimes one has to look at its possibilities, buy some mosquito fabric and put it there, some sticks or what they can, but really the truth is that you need a lot.”

#### Survey

The majority of households used at least one method of insect control in their homes (73%, 226/308). No significant gender differences in perceived effectiveness were observed (χ^2^ = 2.8–7.9 for each method, d.f. = 4, *P*>0.05 for all methods). On the other hand, there were some differences among communities, with a significantly lower proportion of households from Sudzal using mosquito coils and plug-in repellent (χ^2^ = 33.3, d.f. = 2, *P*<0.0001 for both products). However, the use of other measures and products was similar in all three communities (χ^2^ = 3.5–7.5 for other methods, d.f. = 2, *P*>0.05).

**Figure 2:**
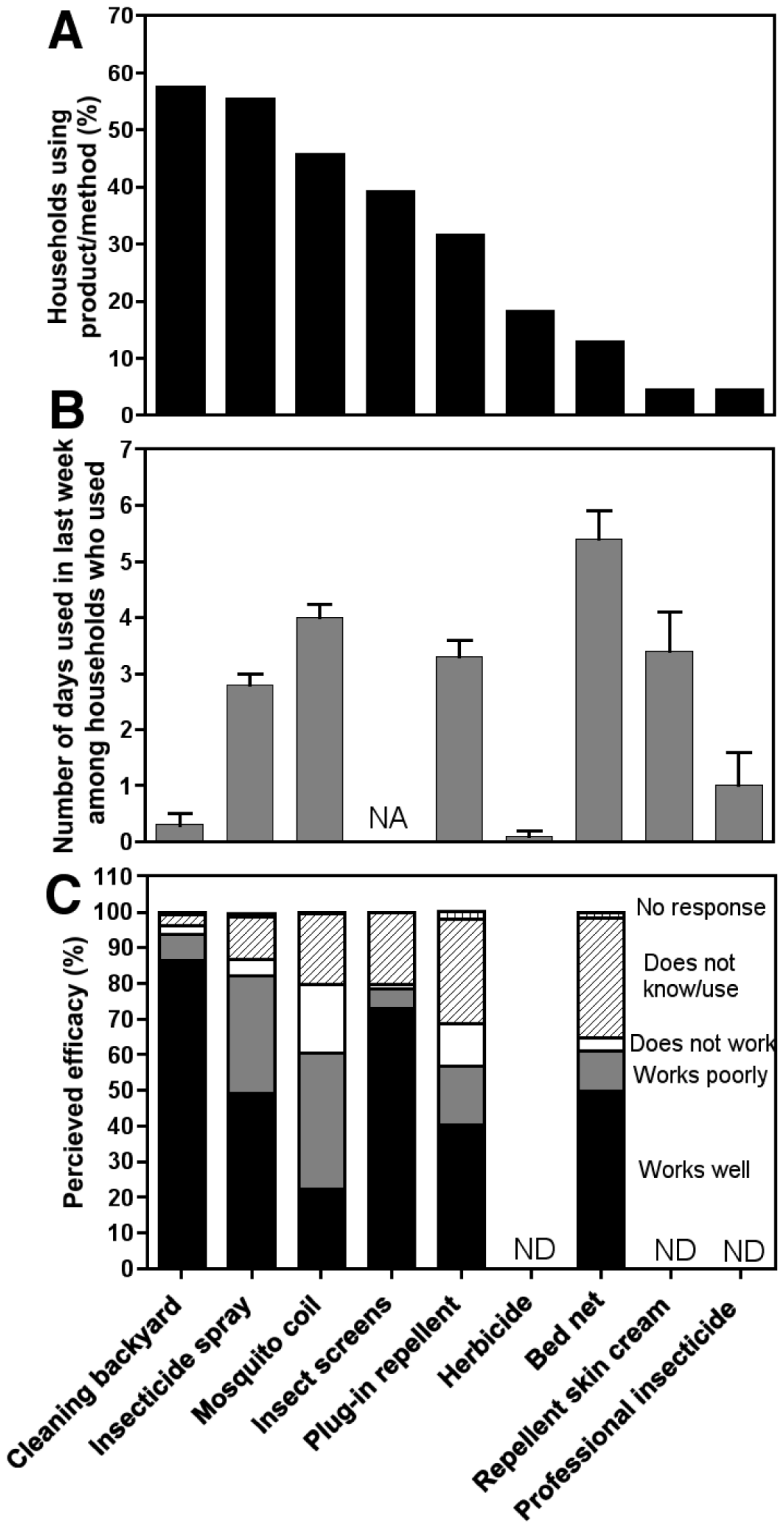
Use and perception of vector control measures. The percentage of households using each product/method (**A**), the number of days each product/method was used in the past week (**B**) and the perceived efficacy of each product/method (**C**) are shown. For perception, products/methods are classified as “works well”, “works poorly”, “does not work”, “does not know/use”, or “no response” as indicated. ND: not determined.

Overall, many households used insecticide aerosol sprays, mosquito coils and plug-in repellent (30–55% of households, Fig. 2A), and used these products 3–4 times a week (Fig. 2B), although a minority of respondents reported these products “worked well” (Fig. 2C). The majority of households reported cleaning their yards (57%, 177/308). However, this was not with great frequency (on average once every 20 days), even though over 86% (204/236) perceived it as an effective measure against insects. Of the minority of households that had insect screens on at least some windows (39%, 92/236), about half (44% (41/92)) had them on all windows. The majority (73%, 172/236) believed screens to be very effective against insects (Fig. 2). Bed or hammock nets were used by a minority of households (13%, 38/292), though these seemed to be considered effective by many households (50%, 117/236). Other control measures such as the use of herbicide in the yard, repellent skin cream, or professional insecticide praying, were either used by few households (<10%), or infrequently (less than once a week), and thus did not seem to be mainstream measures commonly used in the communities (Fig. 2).

**Figure 3:**
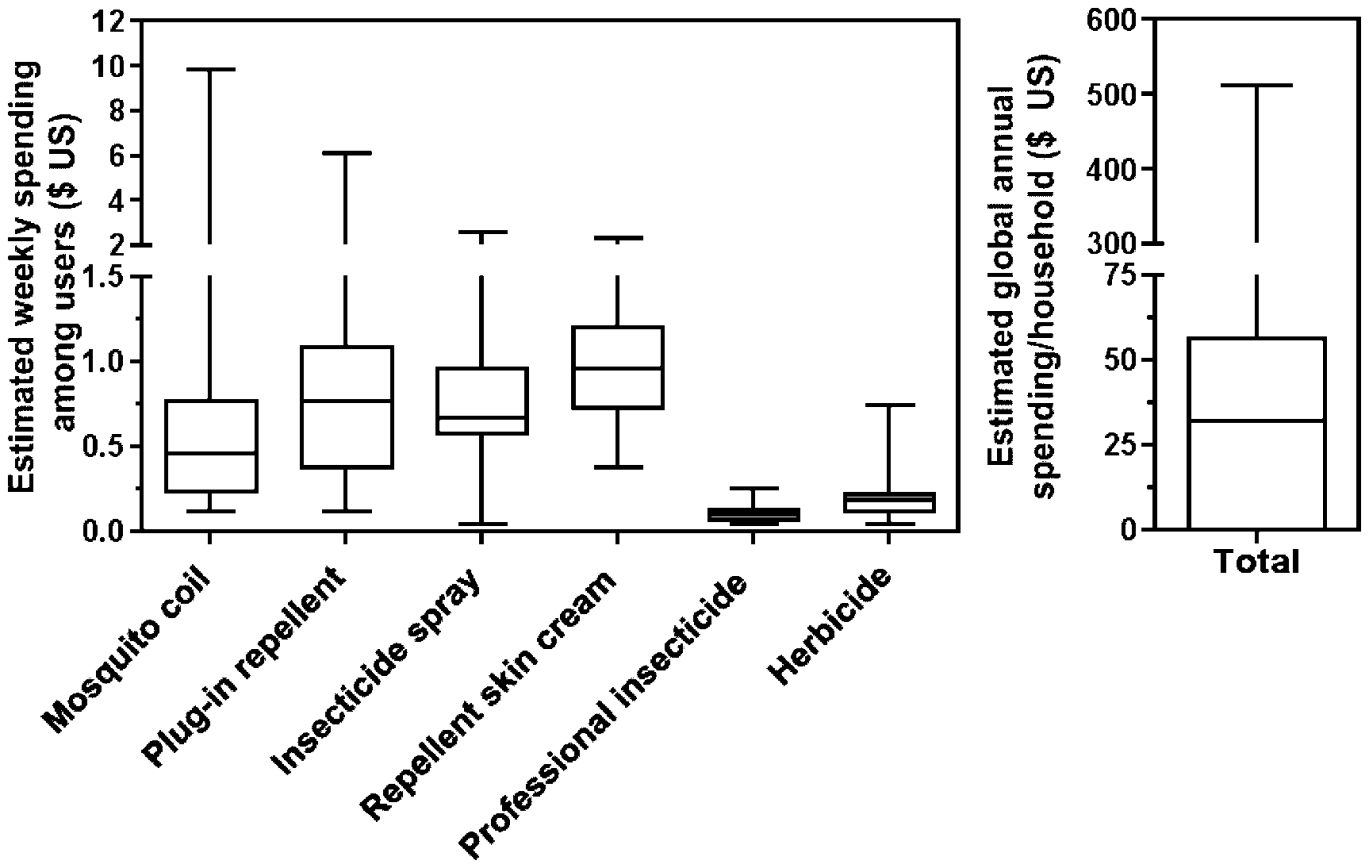
Household estimated spending on vector control products. Number of respondents were N = 107, 57, 126, 9, 9, 32 for mosquito coils, plug-in repellent, insecticide spray, repellent skin cream, professional insecticide and herbicide respectively. Annual spending was calculated based on all 308 households.

When asked about spending on insect control products, households who used mosquito coils, plug-in repellent, repellent skin cream or herbicide reported spending an amount equivalent to between 50 cents to one $ US for one week's supply (Fig. 3). Indeed, mosquito coils and plug-in repellents are bought on a daily or weekly basis, and households had an excellent recall of prices practiced in local stores. Extrapolating from reported spending and usage data, we estimated the median global spending of a typical household to be 32 $ US over the course of a year, although a few households may spend up to a few hundred $ US (Fig. 3).

Unexpectedly, households with window insect screens had a similar use of other products such as insecticide spray, mosquito coils, and plug-in repellent as those without screens ((χ^2^ = 2.8, d.f. = 2, *P*>0.05 for all three products). Spending on these products was also similar between households with and without insect screens. On the other hand, a large majority of households who did not have insect screens on their window reported that they would like to have screens (91%, 131/144), particularly if they were free (98%, 129/131). A much lower proportion said they would be willing to pay for insect screens (64%, 84/131), suggesting that cost is a major barrier for households to have insect screens in their homes.

## Discussion

We present here a first assessment of community knowledge, perceptions and practices related to Chagas disease and triatomine vectors in Yucatan, Mexico, evaluated through mixed qualitative and quantitative approaches. This study provides key data for the design of culturally acceptable vector control interventions and identifies specific needs for Chagas disease education.

Our data demonstrate that community members are aware of triatomines, knowledgeable about their habits, and frequently come into contact with them in their houses. Community members' identification of pictures of adult triatomines, but not nymphs, and their descriptions of bugs flying in through windows at night matches our previous observations and description of the seasonal infestation by adult *T. dimidiata* and its limited colonization of houses [31], [33], [35]–[37]. Community members are also well aware of some specific aspects of triatomine behavior, such as their presence in the bushes surrounding villages, and their habit of hiding in rock piles, which have been identified as risk factors for house infestation [34], [35], [39], [40]. This well-developed knowledge of triatomines is similar to that observed in other communities from endemic regions [22]–[29]. The elevated proportion of households reporting seeing triatomines inside their homes is in agreement with the high infestation by *T. dimidiata* that has been described in the Yucatan peninsula and further illustrates the risk of Chagas disease in the region.

While triatomines are considered “dangerous,” this seems to be due to the actual bite wound rather than a complete understanding of Chagas disease. A few participants did know that a chronic condition can develop over time, but most had a limited understanding of Chagas disease transmission, its relationship with triatomines and the specific health consequences. Again, this appears similar to what has been observed in other countries and communities [23]–[29]. Even those who mentioned a chronic disease always expressed some level of skepticism (e.g. ‘I've been told that…’), indicating that the threat of Chagas disease has not truly been appropriated in these communities and remains theoretical.

This lack of knowledge and awareness of Chagas disease and its relationship with triatomines can be considered a major barrier for vector control, as it likely results in communities having limited interest in and motivation for eliminating triatomines. There is thus an important need for education on these aspects. The health centers of these communities appear to be appropriate settings for information dissemination, although multiple sources and improved educational materials may allow for more extensive and effective diffusion. Indeed, most of the inhabitants referred to talks and pamphlets provided by our research group through the health center over the past years as their main source of information. Although it was not the focus of our study, community members spontaneously and frequently spoke of mosquitoes and dengue fever, and seemed to have a good knowledge of this disease and its prevention, suggesting that dengue vector control and education activities, together with the high visibility of dengue fever (incidence of 280/100,000 inhabitants in the state of Yucatan) may have been effective in raising awareness of dengue fever [50] and could serve as models for Chagas disease interventions. However, the high visibility of dengue fever compared to Chagas disease may partially explain higher community awareness and threat perception. Focus group discussion findings indicate that participants do not have personal experience with Chagas disease. However, many had friends or relatives that had had dengue fever, and were able to discuss the effects of the disease.

Insect control, and more specifically mosquito control, is a concern for a majority of community members, though prevention of triatomine infestation is not a priority. They frequently use a number of domestic insecticide products, including insecticide spray, mosquito coils, and plug-in repellents, even though they are aware of their limited effectiveness. Many spend around one $ US each week to purchase these products, leading to a cumulative spending of about 32 $ US annually and per household. Given that the average income of these households is limited (63% of households benefit from the social welfare program “*Oportunidades*,” [39] which targets families with an estimated income below a poverty threshold established at 600–1300 $US/person/year), this spending supports the importance of vector control for community members. Based on motivation to control insects in general and mosquitoes in particular, it is likely that many households would engage in further vector control efforts directed against triatomines if they recognized them as severe threats. Interventions aimed at preventing triatomine infestation, but that also protect against other insects such as mosquitoes, are likely to be adopted by communities.

Importantly, the studied communities perceive some strategies to be potentially more effective than insecticide products. For example, they stressed the importance of having a clean yard to avoid insects and associate triatomine infestation with a dirty yard. This is in agreement with modeling and field studies which indicate that a clean yard can result in a 50–60% reduction in house infestation by triatomines [14], [41]. Nonetheless, time and effort may remain barriers to yard cleaning. Many yards are infested and even colonized by *T. dimidiata* [36], [38]. Another issue is that yard cleaning is perceived as requiring a collective effort to be effective, and many participants mentioned that their efforts in maintaining a clean yard were ineffective if neighbors did not also maintain their yards appropriately. Because yard cleaning also contributes to mosquito vector control, education messages may be designed to synergize efforts against dengue fever and Chagas disease vectors.

Window insect screens were also perceived as effective, as previously demonstrated by modeling and field studies [14], [15], [41], and desirable by community members. The main obstacle to wider use is cost, as screens are perceived as unaffordable for many households (F9: “Either we put mosquito screens or we eat.”). However, the annual cumulative spending on insecticide products of a typical household is comparable to the price of screens. Indeed, we reported in a previous pilot study a cost of 40–50 $US/house for the manufacture and installation of screens [14]. Appropriate funding schemes might make it possible for households to redirect their current vector control spending from mosquito coils, insecticide spray and plug-in repellent, which have little or no effect on triatomine infestation [39], to more effective insect screens, [14], [15], [41] and thus benefit from better vector control. Such funding or microcredit schemes, together with specific Chagas disease education, may be included as part of the *Seguro Popular* health insurance program, which targets many community members. Again, the effectiveness of insect screens in preventing all vector-borne diseases should be emphasized. Indeed, multi-disease and integrated vector control interventions are believed to be more cost-effective, as well as more effective and sustainable than single disease-centered interventions [51], [52].

Finally, women are mostly responsible for insect prevention in the home, including measures taken in the peridomestic area, and should be engaged in the design and implementation of interventions, as observed in other communities [53]. They are motivated to protect children especially; the benefit of avoiding acute Chagas disease in children should be emphasized in future educational efforts. Though they were not included in the current analysis, we found children to be interested in and enthusiastic about study activities, such as yard cleaning. Involving them in prevention activities might also increase community participation [24], [54].

This study is a formative, descriptive analysis of the knowledge, attitudes and practices of communities related to triatomine vector control and Chagas disease that relies on participant response. Possible limitations include response bias (all methods) and social desirability bias (focus group discussions). In order to minimize response bias, investigators trained in an anthropological approach to qualitative data collection conducted freelisting and ranking exercises as well as focus group discussions. Also, investigators purposefully positioned themselves as non-experts and redirected participant questions about biting insects, insect prevention, and insect-related disease back to participants. Social desirability bias may have caused participants to over report frequency or intensity of insect prevention practices, as these were said to be important for child health and therefore indicative of good parenting. Recall bias may have affected responses to some survey questions, in particular those that asked respondents to quantify prevention method use and price. Finally, the communities in which we worked may be more familiar with triatomines and Chagas disease than surrounding communities because of education sessions in relation to past research, but this only strengthens our conclusion that Chagas disease, though present, is not well understood by communities at risk for infection.

In conclusion, the studied communities in Yucatan, Mexico appear very knowledgeable about triatomine vectors, but there is a clear need for education on Chagas disease to increase awareness of and interest in controlling this disease. Mosquitoes rather than triatomines are a concern for most households, who dedicate a significant proportion of their limited income to the purchase of products of limited effectiveness against triatomines. Alternatively, yard cleaning and window screens are perceived as effective and desirable by community members. These two methods of vector control, which could prevent both triatomines and mosquitoes from entering houses and biting inhabitants, should be promoted. The cost of insect screens, however, is a barrier that should be addressed, potentially through financing schemes. Importantly, synergism with dengue vector control efforts should be leveraged to increase community involvement and ensure sustainability of Chagas disease control.

## Supporting Information

Table S1 **Ranking of insects or bugs that bite people in the village.**
(DOCX)

Table S2 **Relative effectiveness of insect prevention methods.**
(DOCX)

Text S1 **Original quotes in Spanish.**
(DOCX)
