# Supplementary material for: Opportunities for Improved Chagas Disease Vector Control Based on Knowledge, Attitudes and Practices of Communities in the Yucatan Peninsula, Mexico
Source: PLoS Negl Trop Dis. 2014 Mar 27;8(3):e2763. doi: 10.1371/journal.pntd.0002763 (PMC3967964; doi:10.1371/journal.pntd.0002763)
Supplement: Table S1 — Ranking of insects or bugs that bite people in the village. (DOCX) [file pntd.0002763.s001.docx]

| **Supplementary Table S1. Ranking of insects or bugs that bite people in the village* (n=45)** | | |  |
| --- | --- | --- | --- |
|  | Number of respondents (%) | Salience** | |
| Mosquito | 39 (87) | 0.71 | |
| “Pic” | 34 (76) | 0.58 | |
| Scorpion | 31 (69) | 0.43 | |
| Snake (any type) | 26 (58) | 0.24 | |
| Cockroach | 26 (58) | 0.35 | |
| Tarantula | 18 (40) | 0.2 | |
| Horsefly | 17 (38) | 0.19 | |
| Ant (any type) | 16 (36) | 0.21 | |
| Bee | 13 (29) | 0.12 | |
| Wasp | 11 (24) | 0.09 | |
| Tick | 10 (22) | 0.1 | |
| “Chinche” | 8 (18) | 0.09 | |
| * Table shows only the most frequently mentioned insects. | | |  |
| ** Salience = (inverted rank of item/number of items listed)/n | | |  |
