# Supplementary material for: Opportunities for Improved Chagas Disease Vector Control Based on Knowledge, Attitudes and Practices of Communities in the Yucatan Peninsula, Mexico
Source: PLoS Negl Trop Dis. 2014 Mar 27;8(3):e2763. doi: 10.1371/journal.pntd.0002763 (PMC3967964; doi:10.1371/journal.pntd.0002763)
Supplement: Table S2 — Relative effectiveness of insect prevention methods. (DOCX) [file pntd.0002763.s002.docx]

| **Supplementary Table S2. Relative effectiveness of insect prevention methods (n=44)** | |
| --- | --- |
| Average rank | Method |
| 1 | Aerosol insecticide |
| 2 | Patio cleaning |
| 3 | House cleaning |
| 4 | Window/door screens |
| 5 | Mosquito coil |
| 6 | Plug-in mosquito repellent |
| 7 | Bed/hammock net |
| 8 | Brush/plant burning |
| 9 | Herbicide |
| 10 | Insect repellent |
