# Supplementary material for: Opportunities for Improved Chagas Disease Vector Control Based on Knowledge, Attitudes and Practices of Communities in the Yucatan Peninsula, Mexico
Source: PLoS Negl Trop Dis. 2014 Mar 27;8(3):e2763. doi: 10.1371/journal.pntd.0002763 (PMC3967964; doi:10.1371/journal.pntd.0002763)
Supplement: Text S1 — Original quotes in Spanish. (DOCX) [file pntd.0002763.s003.docx]

**Supplementary text S1.**

Original quotes in Spanish

M1. “Bajo las piedras en el monte. También en las casas donde hay mucha basura… bajo las piedras. si donde hay muchas basuras, carton.”

F1. “Si, por ejemplo es que recogemos las piedras y lo amontonamos y a veces ellos ahí hace su casita, ahí vive.”

F2. “El *pic* es por las noches, porque en las noches es cuando entra a picar.”

M2. “Es malísimo, el *pic* es malísimo, transmite no sé qué, una enfermedad...”

M3. “Al momento que te pica queda hinchado y te dura... y al tiempo es la reacción, al instante no se ve, no se ve si te puede dar alguna enfermedad, pero ya después al paso del tiempo sí, se detecta la enfermedad, dicen que del corazón.”

F3. “Que produce paro cardiaco, eso dicen, eso escuche en la plática”.

F4. “Porque una vez que te pique siempre te va a dar el Chagas, porque la Chagas dicen que es un microbio que no lo ves.”

F5. “Dicen que te dañaba el corazón y todos tus órganos, los envejecía, que así te mataba, absorbe la sangre de tu corazón y dicen que se llama Chagas la enfermedad.”

F6. “Yo lo fumigo, mayormente lo fumigo porque caen toditos, no se quedan.”

M4. “Deshierbar, como por ejemplo las hierbas, los cacharros, las piedras, juntarlos para que no tenga donde esconderse los bichos.”

M5. “Casi todos los bichos, porque cuando ven limpio no les gusta, les gusta donde hay basura.”

M6. “Mosquiteros. Si beneficia mucho porque si hay calor puedes abrir tu puerta y no entran los moscos.”

F7. “Para mí el mejor, es tener miriñaques, porque eso si evitan que entren ... y tener limpio lo que es el terreno.”

F8. “O ponemos miriñaques o comemos.”

F9. “Pero a veces no hay la economía para poner miriñaques como decía la señora X, a veces lo pone uno a su posibilidad, compra uno la malla y le pone ahí, palitos o lo que se pueda pero pues si la verdad si se necesita mucho.”
